# Silencing of XB130 Is Associated with Both the Prognosis and Chemosensitivity of Gastric Cancer

**DOI:** 10.1371/journal.pone.0041660

**Published:** 2012-08-23

**Authors:** Min Shi, Weizhen Huang, Li Lin, Dayong Zheng, Qiang Zuo, Lin Wang, Nina Wang, Yajun Wu, Yulin Liao, Wangjun Liao

**Affiliations:** 1 Department of Oncology, Nanfang Hospital, Southern Medical University, Guangzhou, China; 2 Department of Cardiology, Nanfang Hospital, Southern Medical University, Guangzhou, China; Hong Kong University of Science and Technology, China

## Abstract

XB130 is a newly characterized adaptor protein that was reported to promote thyroid tumor growth, but its role in the progression of other kinds of cancer such as gastric cancer (GC) remains unknown. Accordingly, we investigated the association between XB130 expression and the prognosis of GC patients. The subjects were 411 patients with GC in stages I to IV. XB130 expression was examined in surgical specimens of GC. Kaplan-Meier analysis and the Cox proportional hazards model were used to assess the prognostic significance of XB130 for survival and recurrence. Moreover, GC cells stably transfected with XB130 short hairpin RNA were established to analyze the effect of XB130 on sensitivity of chemotherapy. The results show that both XB130 mRNA and protein expression were detectable in normal gastric tissues. The overall survival time of stage IV patients and the disease-free period after radical resection of GC in stage I–III patients were significantly shorter when immunohistochemical staining for XB130 was low than when staining was high (both *p*<0.05). XB130 expression also predicted tumor sensitivity to several chemotherapy agents. Viability of both XB130-silenced SGC7901 cells and wild-type cells was suppressed by 5-fluorouracil (5-FU), cisplatin, and irinotecan in a dose-dependent way, but cisplatin and irinotecan were more sensitive against sXB130-silenced GC cells and 5-FU showed higher sensitivity to wild-type cells. When treated by 5-FU, patients with high expression of XB130 tumors had a higher survival rate than those with low expression tumors. These findings indicate that reduced XB130 protein expression is a prognostic biomarker for shorter survival and a higher recurrence rate in patients with GC, as well as for the response to chemotherapy.

## Introduction

Oncogenes and anti-oncogenes have a vital role in the development and progression of Gastric cancer (GC), and genetic heterogeneity has been proven to influence the prognosis markedly. Therefore, hunting for novel genes and proteins with potential value as diagnostic or prognostic tools is important, and targeting novel oncogenes is another promising approach to cancer therapy.

XB130 is a newly identified adaptor protein that is strongly expressed in the spleen and thyroid of humans, while it shows weak expression in the kidney, brain, lung, and pancreas [1]. XB130 has been detected in follicular and papillary thyroid carcinoma, as well as in human lung carcinoma cell lines [2]. Although its expression was reduced in thyroid carcinoma, XB130 was found to be a tumor promoter [2]. It has been reported that XB130 not only has a role in cell proliferation, survival, motility, and invasion [2], [3], [4], but is also involved in signal transduction [1]. XB130 is regulated by Rac and by the cytoskeleton [3], and it is involved in the activation of c-Src [1] and the phosphatidyl-inositol-3-kinase (PI3K)/Akt pathway [4], which in turn regulate cytoskeletal function [3]. These signaling pathways have also been shown to have an essential role in the development and progression of GC [5], [6], [7]. Hence, there could be a role of XB130 in GC too. However, no research on this newly found adaptor protein has been done in the field of gastroenterology so far. In this study, we hypothesized that XB130 expression might be associated with survival and/or tumor recurrence as well as with the chemosensitivity of GC.

## Results

### XB130 expression in normal gastric tissues and GC

**Figure 1:**
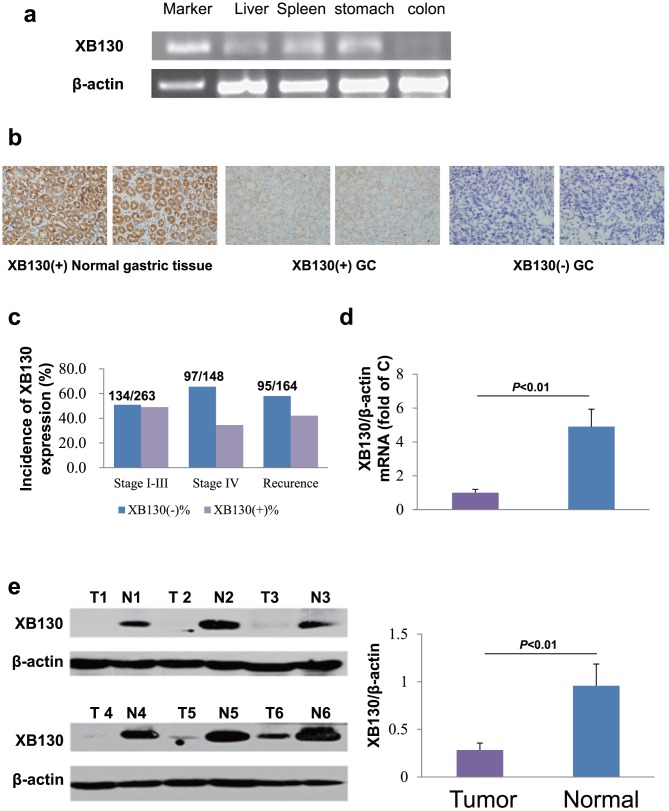
XB130 protein or mRNA expression in gastric tissues. (**a**) The XB130 gene is expressed in the human liver, spleen, colon and stomach. (**b**) Representative images of XB130 protein immunostaining in GC tissue and the adjacent non-tumor tissue. (**c**) Incidence of XB130-negative and XB130-positive GC in stages I–III (n = 263) and stage IV (n = 148). (**d**) In stage I–III GC after radical resection, XB130 mRNA expression was significantly lower in tumor tissue than in the corresponding adjacent non-tumor tissue quantified by fluorescence PCR *(p*<0.01, n = 9 per group*)*. (**e**) Expression of XB130 protein by stage I–III GC (T) is significantly reduced compared with that by normal gastric tissue (N) on western blotting (*p*<0.01, n = 6 per group). Samples in (a), (d) and (e) were fresh tissues from patients with stage I–III GC who received radical resection surgery, while samples in (b) and (c) were from patients with stage I–IV GC as described in the method section.

Different from previous reports, we confirmed that XB130 is constitutively expressed in normal tissues of human liver, colon, spleen and stomach (Figure 1a). Immunohistochemistry was performed to evaluate XB130 expression in all 411 pairs of paraffin-embedded sections from GC and their adjacent non-tumor tissues. We found that XB130 was predominantly expressed in cytoplasm in the normal gastric tissue, while it was significantly downregulated in the tissues of GC. We further categorized the GCs into XB130 positives (high expression with score ≥3) and negatives (low expression with score <3) by the staining scores (Figure 1b). Incidence of XB130 positives and negatives in stage I–III GC were of no difference, but the portion of XB130 negatives was higher in stage IV and in the post-surgery recurrence groups (Figure 1c). Quantified mRNA level of XB130 was detected in stage IV palliative resection samples and significantly reduced in advanced GCs (Figure 1d). Similarly, reduced protein expression of XB130 in advanced GC was also verified by western blot (Figure 1e).

### Low expression of XB130 is correlated with poor prognosis

**Figure 2:**
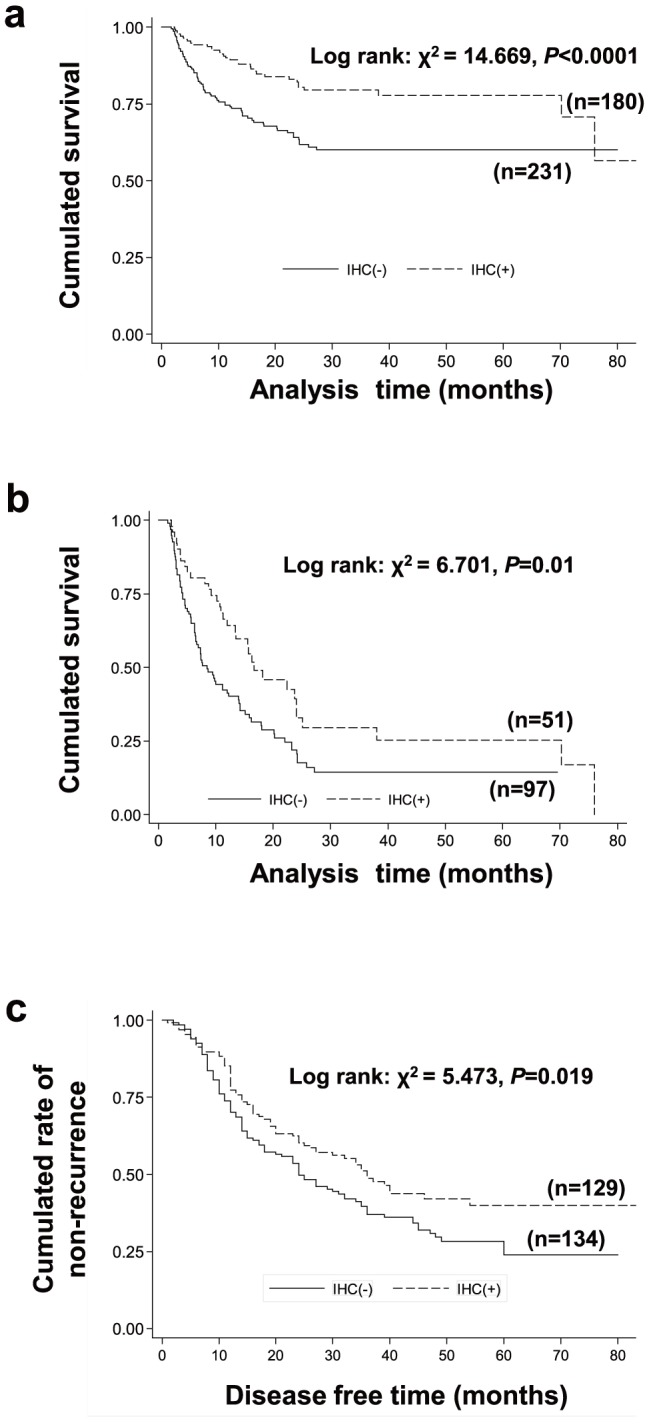
XB130-negative immunostaining predicts a poor prognosis in patients with GC. (**a**) In all 411 patients with GC (stages I–IV), the cumulative survival rate of the XB130-negative group was significantly lower than that of the positive group (*p*<0.0001). (**b**) In 148 patients with stage IV GC, the XB130-negative group had a significantly lower cumulative survival rate than the positive group (*p* = 0.01). (**c**) In patients with stage I–III GC treated by radical resection, the cumulative disease-free survival was significantly lower for the XB130-negative group than the positive group (*p* = 0.019).

In the analysis of overall 411 cases of GC at stage I–IV, cumulated survival rate of patients with XB130 negative staining was significantly lower than the ones with positive staining (Figure 2a). In patients with stage IV GC who lost the surgery cure opportunity, it showed significant lower survival rate in XB130 negative group (Figure 2b). Median overall survival time of patients in advanced stage was longer in XB130 positive group than in negative group (16.7 vs. 8.5 months, HR for negative group was 1.72, p = 0.011; Table 1). In those patients at stage I–III GC who received radical resect surgery, the disease-free survival in XB130 positive group was higher than the negative group (p<0.05; Figure 2c), while the median time to reoccurrence was 36 and 24 months in positive and negative groups, respectively (Table 1, HR for negative patients was 1.2, p = 0.022). These findings indicate that low expression of XB130 is associated with shorter survival and higher recurrence in GC patients.

**Table 1:** Relationship between **c**linicopathological characteristics and survival or recurrence among 411 patients with stage I–IV gastric cancer.

Feature	N (%)	Median (months)	HR (95% CI)	p value
Survival in stage IV GC				
Age			1.38 (0.93–2.03)	0.107
<55 years	64 (43%)	15.1		
≥55 years	84 (57%)	9.7		
Gender			1.05 (0.71–1.57)	0.803
Male	97 (66%)	12.0		
Female	51 (34%)	12.0		
XB130 expression			1.72 (1.13–2.62)	0.011
Negative	97 (66%)	8.5		
Positive	51 (34%)	16.7		
CEA			0.43 (0.29–0.65)	0.000
<5 µg/L	106 (72%)	15.6		
≥5 µg/L	42 (28%)	5.3		
Chemotherapy			2.304 (1.51–3.52)	0.000
No	37(25%)	5.3		
Yes	111(75%)	15.1		
Ascites			0.31 (0.203–0.48)	0.000
No	110 (74%)	15.7		
Yes	38 (26%)	3.8		
Recurrence in stage I–III GC				
Age			1.07 (0.78–1.46)	0.694
<55 years	159 (61%)	35.0		
≥55 years	104 (39%)	27.0		
Gender			1.00 (0.85–1.18)	0.998
Male	177 (67%)	32.0		
Female	86 (33%)	30.0		
XB130 expression			1.20 (1.03–1.40)	0.022
Negative	134 (51%)	24.0		
Positive	129 (49%)	36.0		
Stage				0.000
I	43 (16.4%)		0.18 (0.10–0.34)	0.000
II	89 (33.8%)	36.0	0.57 (0.41–0.79)	0.001
III	131 (49.8%)	18.0		

### Prognostic value of other clinicopathological parameters

**Figure 3:**
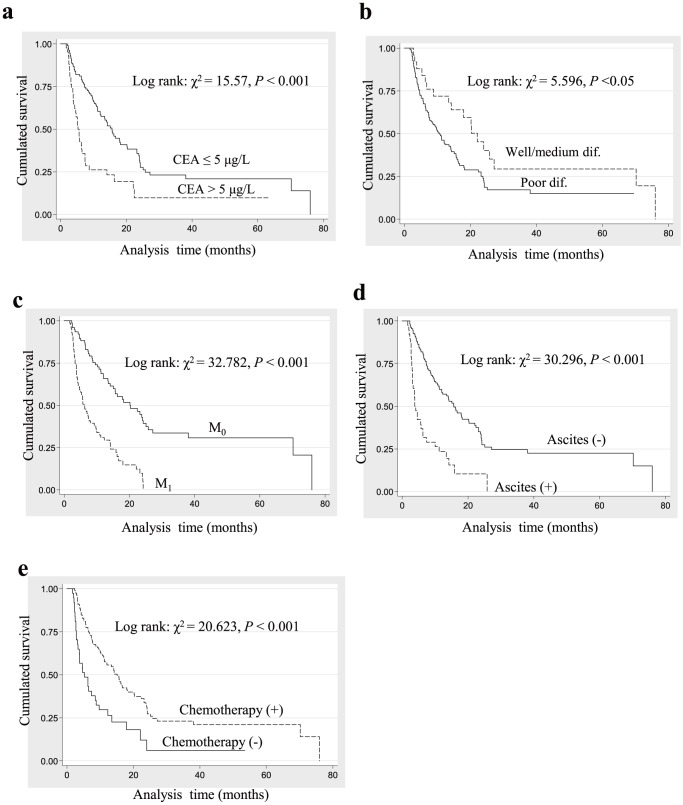
Survival curves for patients with gastric cancer (GC) at the Nanfang Hospital with clinicopathological factors. The figures are labeled as follows: cumulative survival of patients with GC with (**a**) Carcinoembryonic antigen (CEA)≤5 µg/L or >5 µg/L, (**b**) different differential (dif.) degree, (**c**) with (M_1_) or without (M_0_) metastasis, (**d**) with or without ascites, (**e**) with or without chemotherapy.

The influence of other clinicopathological parameters on overall survival and disease-free survival rate was listed in table 1 and figure 3. In addition to XB130, results of Kaplan-Meier survival function analysis in patients at IV stage grouped by the well-known factors such as carcinoembryonic antigen (CEA) concentration, differential degree, metastasis, ascites and chemotherapy demonstrated significant influence on the cumulated survival (Figure 3 a–e). In a univariate Cox regression analysis in patients received radical therapy, we noted that stage was also a significant prognostic factor for reoccurrence (Table 1).

In a multivariate analysis using Cox model, we noted that XB130, CEA, chemotherapy and ascites (all p<0.01) were independent factors to predict HR for death in patients at stage IV, while XB130 (p = 0.044) and stage (p<0.000) were independent factors to predict HR for reoccurrence after radical resection of GC in patients at stage I–III (Table 2).

**Table 2:** Multivariate analysis for overall survival or recurrence.

Covariates	HR	95% CI	P
Survival for stage IV patients			
Age (vs. <55 years)	1.100	0.733–1.652	0.645
Gender (vs. female)	0.983	0.645–1.498	0.936
XB130 (vs. IHC positive)	1.789	1.168–2.739	0.007
CEA (vs. >5 µg/L)	0.477	0.309–0.739	0.001
Chemotherapy (vs. yes)	1.981	1.285–3.054	0.002
Ascites (vs. yes)	0.374	0.241–0.582	0.000
Recurrence for stage I–III patients			
Age (vs. <55 years)	1.004	0.731–1.378	0.983
Gender (vs. female)	0.977	0.703–1.357	0.889
XB130 (vs. IHC positive)	1.379	1.008–1.888	0.044
Stage (vs. stage III)			
Stage I	0.188	0.100–0.353	0.000
Stage II	0.544	0.388–0.763	0.000

Stepwise regression, Wald method. HR: Hazard ratio, CI: confidence.

interval, CEA: carcino-embryonic antigen, IHC: immunohistochemistry.

### Chemotherapeutics sensitivity in response to XB130 silencing

**Figure 4:**
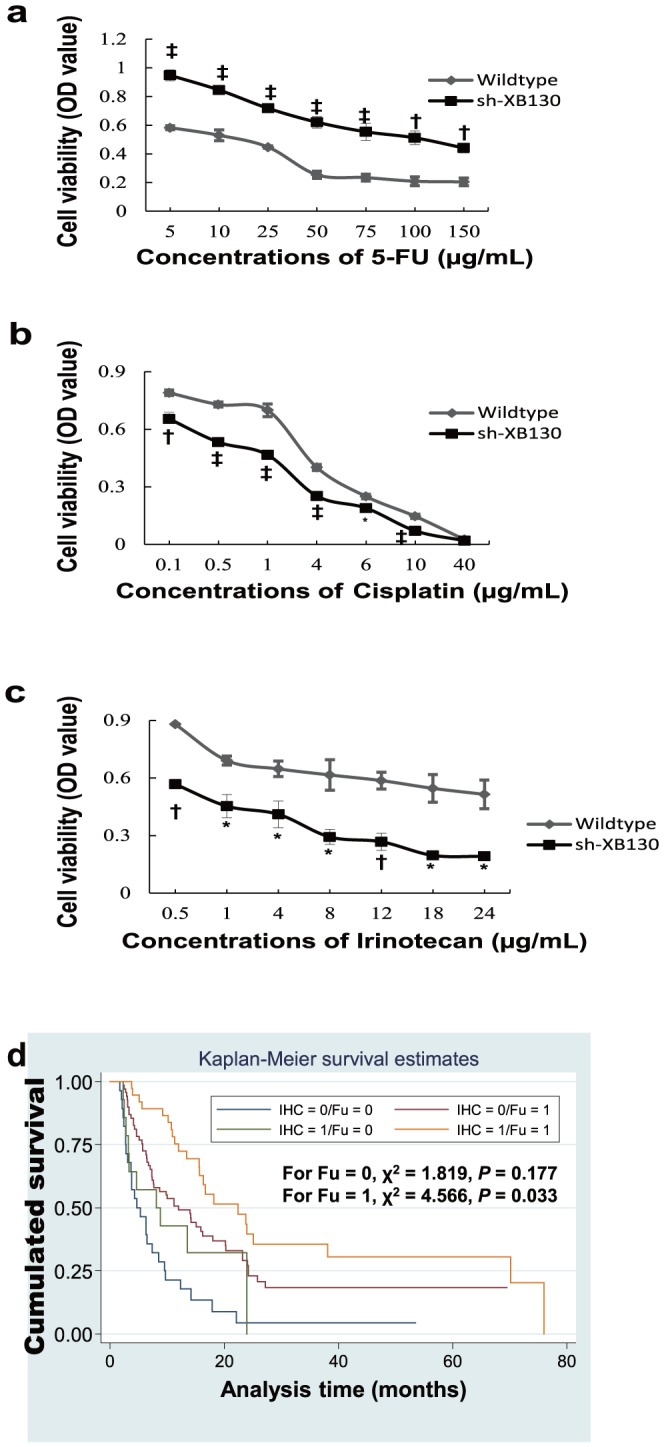
Sensitivity of cultured SGC7901 cells to chemotherapy agents and therapeutic effect of 5-FU in patients with stage IV GC. (**a**) Cells exposed to 5-FU showed better viability in the sh-XB130 group than in the scramble group (n = 3–4 at each concentration in each group). (**b**) Cell viability in the sh-XB130 group is dramatically reduced by exposure to cisplatin (n = 5–10 at each concentration in each group). (**c**) Irinotecan also reduced cell viability in the sh-XB130 group (n = 3–4 at each concentration in each group). ^*^ *p*<0.05, ^†^ *p*<0.01, ^‡^ *p*<0.001 vs. wild-type cells at the corresponding concentration. (**d**) XB130-negative patients had a lower survival rate when treated by 5-FU (Fu = 1).

In order to find out the therapeutic potential in targeting XB130, we assessed cell viability of sh-XB130 in response to 5-FU, cisplatin and irinotecan. The cell viability in sh-XB130 and wildtype groups were both suppressed by all three chemotherapeutic agents in a dose dependent way. Respectively, compare with sh-XB130, wildtype control showed more sensitive to 5-FU manifested by a significant lower cell viability (Figure 4a), whereas cisplatin (Figure 4b) and irinotecan (Figure 4c) were demonstrated to be more effective in sh-XB130 group than in wildtype control.

### Survival analysis in GC patients receiving 5-FU therapy

Sorted by XB130 negative and positive, we evaluated 5-FU therapy in the stage IV GC patients in a retrospective study. Treated by 5-FU, patients with XB130 positive staining enjoyed a higher survival rate than the negative group (Figure 4d). Correspondingly, XB130 positives with 5-FU therapy had the longer median overall survival time (Figure 4d). Since there were few patients treated with cisplatin or irinotecan, the influence of XB130 expression level on clinical therapeutic effects were not compared in present study.

## Discussion

XB130 is a recently cloned 130 kDa-adaptor protein and is reported to be predominantly expressed in the thyroid and the spleen tissues validated by northern blot and plays a multifunctional role in cell survival, proliferation, invasion in thyroid tumor [2], [3], [4], but its mRNA and protein expression in other tissues has not been confirmed by real-time PCR, western blot or immunohistochemistry. In this article, we firstly gave evidence that XB130 mRNA and protein were also constitutively expressed in normal gastric tissue and relatively lower expression in GC cells. These findings are in contrast with previous reports that XB130 mRNA was barely detectable in other tissues except thyroid and spleen [1], [4], partly because they only used northern blot for confirmation. XB130 expression is reduced in thyroid tumor, but it remains completely unclear whether XB130 is a prognostic biomarker of other malignant tumors such as gastric cancer (GC) [2]. In order to confirm that XB130 takes part in the GC progression, we had analyzed survival or recurrence in 411 patients with GC and noted that the XB130 low expression predicted a lower survival and higher recurrence. Furthermore, Chemotherapeutic sensitivity basing on XB130 expression was evaluated for the first time, and cellular experiments and clinical retrospective study indicated that downregulation of XB130 increases drug sensitivity to cisplatin and irinotecan and decreases drug sensitivity to 5-fluorouracil.

Given the carcinogenicity in previous reports [2], [8], [9], XB130 should be regarded as an oncogenic other than a tumor-suppressive protein, although further studies are needed to test whether it is also the case in GC. Then, how to explain the clinical significance of our clinical observation that XB130 downregulation predicts a low overall survival in advanced GC and higher recurrence rate in patients treated by radical resection surgery? In fact, it is known that tumorigenesis can result from alterations of multiple genes and proteins, while XB130, the adaptor protein with more than one functional domain, can be affected by multiple upstream and downstream factors. Therefore, it is too premature to judge whether a gene or a protein is oncogenic or not just basing on its expression in clinical specimens, although the latter did hint some clues. Actually, downregulation of XB130 in GC may be considered a compensatory adjustment, because some tumor suppressors would be mobilized to resist XB130 during the progression of GC. Similarly, it was reported that the tumor suppressor p53 expression was higher in poorly-differentiated GC than in well-differentiated ones [10], while in early gastric cancers with low levels of apoptosis, increased expression of Bcl-2 and p53 was more likely to promote metastasis [11], [12].

XB130 may be a proto-oncogene, which preserves in normal tissue. Such proto-oncogenes may contribute to maintain physical functions of cell renewal and upward migration in normal stomach. Meanwhile, there is another set of regulating genes that prevent normal cell over-proliferation and metaplasia. However, once the microecological balance is disequilibrated, cell proliferation and metastasis is out of control, consequently leading to carcinogenesis [13]. This classical theory of proto-oncogene may give an acceptable explanation why normal tissue with such high XB130 expression still remains “healthy”. It is known that many genes and signaling pathways play pro-oncogenic or anti-oncogenic roles on a context-dependent manner, so it is not uncommon that the same gene may exert different effect at different stages or in different tissue types of cancer development [14], [15], [16]. In the present study, although we did not touch upon the functions of XB130 in normal gastric tissue, this topic is interesting as well and need further research.

If XB130 expression pattern can serve as a surrogate marker predictive of chemotherapy response, it would also provide an explanation for the prognosis. Currently, fluoropyrimidine derivatives-based and platinum compound-based combination regimens have been accepted as conventional first line treatment for GC [17], while irinotecan, a topoisomerase I inhibitor, is employed as the second-line treatment [18], and cisplatin has been largely used in the treatment of advanced, unresectable GC [19]. In the present research, chemotherapeutic-sensitivity studies basing on XB130 expression level were carried out. In GC cells, XB130 knockdown indicated better responsiveness to cisplatin and irinotecan, but with less sensitivity to 5-FU. Our clinical data showed a shorter overall survival time in 5-FU-treated patients with low expression of XB130, suggesting that XB130 downregulation reduces the responsiveness to 5-FU, which may be another explanation for the poor prognosis in patients with low expression of XB130 in this study. Our findings in GC cell line implicate that in advanced GCs, most of which are featured by XB130 low expression, may benefit more from cisplatin and irinotecan other than 5-FU. Given the heterogeneous genomic background such as the different expression of XB130 in GC, it is possible that some populations might benefit from irinotecan and/or cisplatin as it suggested in our cellular experiment, and is worthwhile for further investigation. Further prospective investigations may determine whether XB130 expression patterns can be employed to help stratify patients into different multimodal treatment regimens.

In summary, our present study has provided first evidence that XB130 existence in gastric tissue and GC for the first time. We verified that chemotherapeutic sensitivity evaluation basing on XB130 expression was firstly directed, indicating that XB130 low expression patients might be sensitive to cisplatin and irinotecan, yet senior evidence requires. The clinical prospective of this study includes (1) XB130 may act as GC prognostic biomarker for its low expression implicating for unfavorable outcomes; (2) Since XB130 low expressed GC are responsive to cisplatin and irinotecan superior to 5-fluorouracil in our chemotherapeutic sensitivity study, assessment of XB130 expression may help guide clinical medication in GC.

## Materials and Methods

### Patients and tissue specimens

All 411 patients were histologically diagnosed at Nanfang Hospital, Southern Medical University (Guangzhou, Guangdong, China) from 2000 to 2011. Tumor stage was defined according to the 7th edition of the AJCC cancer staging manual 2010. Samples for diagnostic purposes were taken with the consent from each patient. The study was approved by the Institutional Review Board of the Nanfang Hospital, Southern Medical University. The mean follow-up time for all patients was 59.5 (95% CI: from 55.5 to 63.5) months. The clinical characteristics and XB130 expression of all the GC patients are described in Table 1. Patients of I–III stage GC were performed radical resection, while patients at stage IV received palliative operation.

Prior to immunohistochemistry analysis, the paraffin-embedded primary tissue specimens were cut into 4 µm-thick sections, and mounted on glass slides. Nine pairs of tumor and cancer-adjacent normal tissues from stage IV GC patients were randomly collected for quantitative real-time PCR and six pairs for western blot analysis.

### Immunohistochemistry

Immunohistochemical staining was carried out using the Dako Envision System (Dako, Carpinteria, CA) and rabbit anti-XB130 Ab (1∶100; Abnove) following the manufacturer's recommended protocol. For XB130 assessment, the tissue section was scanned entirely to assign the scores. The staining intensity was scored as 0 (negative), 1 (weak), 2 (medium), or 3(strong). The extent of staining was scored as 0 (0%), 1 (1%–25%), 2(26%–50%), 3 (51%–75%), or 4 (76%–100%), according to the percentages of positively stained areas in relation to the whole carcinoma area (or entire section for normal samples). The sum of the staining intensity and extent scores was used as the final staining scores (0–7) for XB130. For the purpose of survival evaluation, tumors having a final staining score of ≥3 were considered to be positive. XB130 immunostaining was evaluated independently by two individuals blinded to the clinical parameters.

### Fluorescence quantitative PCR

Total RNA was extracted using Trizol kit (Invitrogen, Carlsbad, CA, USA). Reverse transcription of cDNA was then obtained with iScript™ cDNA Synthesis Kit (Bio-Rad Laboratories, Hercules, CA, USA) following manufacturer's instructions. All RNA data were presented relative to the housekeeping gene β-actin using the ΔΔCt method. Routine PCR was also performed to examine the expression XB130 in different organ of human and mouse (primer sequences in Table S1).

### Western blotting

Tissues were washed twice with cold phosphate-buffered saline (PBS) and lysed with protein lysis buffer for 30 min. Centrifugation was performed, and protein-containing supernatant was retained. The protein lysates were separated electrophorectically on 10% SDS-polyacrylamide gel and transferred onto polyvinylidene fluoride membranes (Immobilon P, Millipore, Bedford, MA). Then, immunoblotting was performed by using rabbit anti-XB130 antibody (PradoWalnut, CA, USA) and β-actin (Santa Cruz, CA). Immunoreactive bands were visualized by the enhanced chemiluminescence method (Amersham) with a western blotting detection system (Kodak Digital Science, Rochester, NY, USA) and were quantified by Image software QuantityOne v4.6.2.

### Cell culture

Cells from SGC7901 cell line were cultured in complete medium [Roswell Park Memorial Institute (RPMI) 1640 medium (Invitrogen, Carlsbad, CA, USA) with 10% fetal bovine serum (FBS) (Hyclone)]. Stably-transfected cells were maintained in media with the presence of puromycin (Sigma-Aldrich). Cells were incubated at 5% CO_2_ at 37°C.

### Stably-transfected cell lines establishment

Three different shRNA sequences of XB130 were cloned into pSuper-Rretro-puromycin plasmid with restriction enzyme Bgl II, Hind III (New England Biolabs) and T4 DNA ligase (Takara). Both pSuper-Rretro-puro-shXB130 and pSuper-Rretro-puro were constructed. pSuper-Rretro-puromycin-shXB130 combined with packing plasmid vector or scramble vector as negative control were packed into virus using the calcium phosphate method. SGC7901 cells were transfected by shRNA plasmids using Lipofectamine 2000 (Invitrogen, Carlsbad, CA, USA), repeated three times. Cells were cultured as mentioned above, and single colonies were chosen by western blotting and fluorescence quantitative PCR and used for further experiments. Three shRNA targeting XB130 and one scramble were designed (Table S2). Both mRNA and protein expression of XB130 (primer sequence in Table S1, expression results in Figure S1A, B) were confirmed by real-time PCR and western blot. Adenovirus infection efficiency was showed in Figure S1C and D.

### Cell viability analysis

Trypsinized and seeded on 96-well plates at initial density of 0.2×10^4^/well, cells were cultured and observed at 1, 3, 5 and 7 day. Cell viability was determined by the methyl thiazolyltetrazolium (MTT) assay according to the manufacturer's instructions. The absorbance was measured at 570 nm, with 655 nm as the reference wavelength. All experiments were performed in triplicates.

### Chemotherapeutics-sensitivity assessment in GC cells

Cells of wildtype control and sh-XB130 were cultured in the medium with 5-fluorouracil (5-FU), cisplatin or irinotecan by different concentrations. Forty eight hours later, cell viability was evaluated.

### Statistical analysis

Results are reported as the mean ± SEM or median. The chi-square test for categorical variables and Student's t-test for continuous variables were used. Survival and recurrence rates were calculated according to the Kaplan–Meier method. Statistical significance was accepted at a *p* value of less than 0.05.

## Supporting Information

Figure S1 Validation of the gene silencing effect of small hairpin RNAs of XB130 (sh-XB130) and the infective efficiency of adenovirus. XB130 downregulated cells line models were confirmed by real-time PCR (**a**) and Western blot (**b**). Cells transfected by scramble vector served as negative control and non-transfected MOCK as control. The infective efficiency of adenovirus in 293FT cultured cells was confirmed by normal (**c**) and fluorescence (**d**) microscopies. Inset in A is the amplification curve of real-time PCR.
(PPT)

Table S1 Primer sequences for Real-time or routine PCR.
(DOC)

Table S2 XB130 Sh-RNA sequences.
(DOC)
