# Supplementary material for: Silencing of XB130 Is Associated with Both the Prognosis and Chemosensitivity of Gastric Cancer
Source: PLoS One. 2012 Aug 23;7(8):e41660. doi: 10.1371/journal.pone.0041660 (PMC3426513; doi:10.1371/journal.pone.0041660)
Supplement: Figure S1 — Validation of the gene silencing effect of small hairpin RNAs of XB130 (sh-XB130) and the infective efficiency of adenovirus. XB130 downregulated cells line models were confirmed by real-time PCR (a) and Western blot (b). Cells transfected by scramble vector served as negative control and non-transfected MOCK as control. The infective efficiency of adenovirus in 293FT cultured cells was confirmed by normal (c) and fluorescence (d) microscopies. Inset in A is the amplification curve of real-time PCR. (PPT) [file pone.0041660.s001.ppt]

## Slide 1
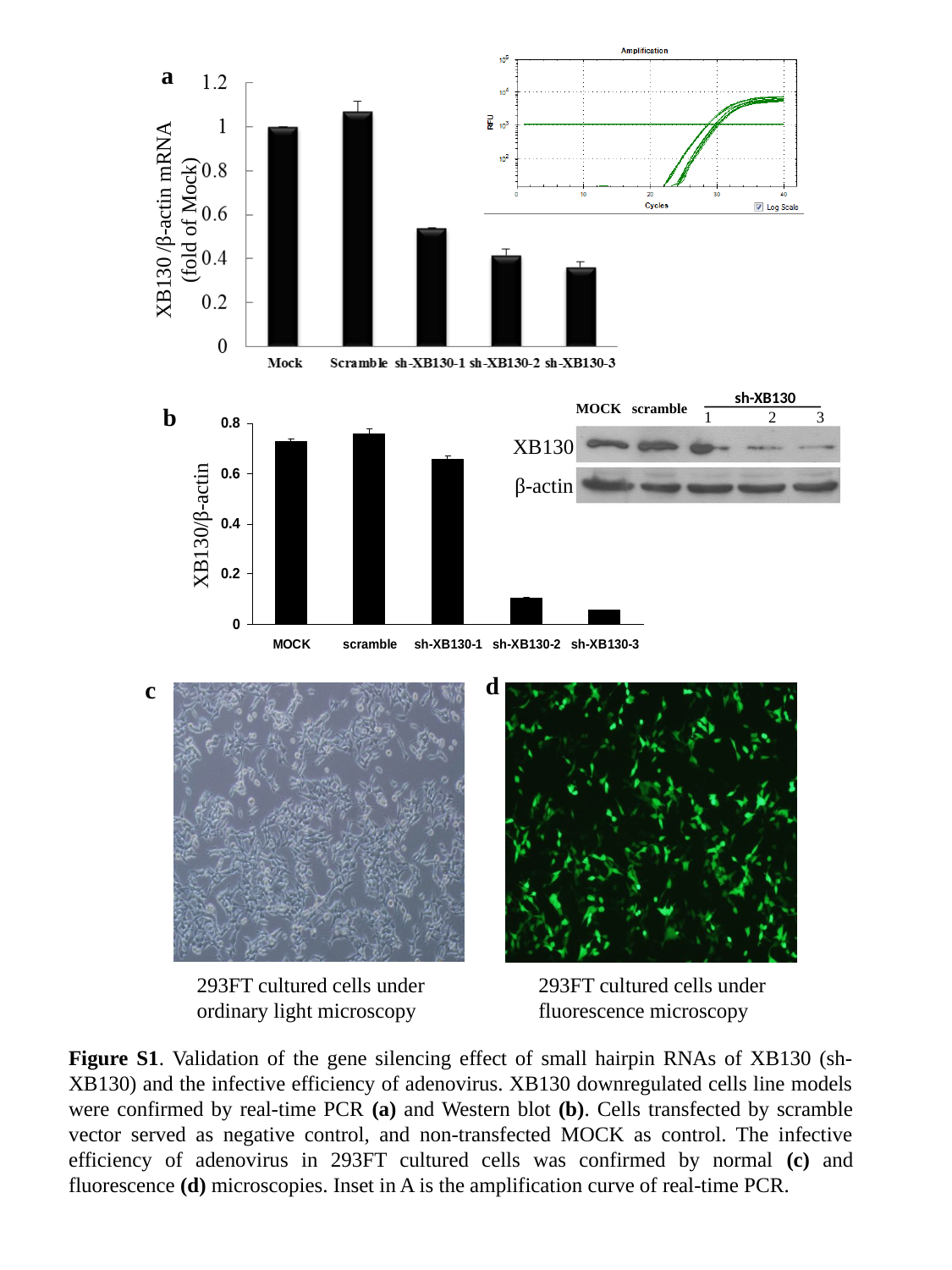

a
XB130 /β-actin mRNA (fold of Mock)
sh-XB130
MOCK scramble
1 2 3
XB130
β-actin
b
XB130/β-actin
d
c
293FT cultured cells under ordinary light microscopy
293FT cultured cells under fluorescence microscopy
Figure S1. Validation of the gene silencing effect of small hairpin RNAs of XB130 (sh-XB130) and the infective efficiency of adenovirus. XB130 downregulated cells line models were confirmed by real-time PCR (a) and Western blot (b). Cells transfected by scramble vector served as negative control, and non-transfected MOCK as control. The infective efficiency of adenovirus in 293FT cultured cells was confirmed by normal (c) and fluorescence (d) microscopies. Inset in A is the amplification curve of real-time PCR.
