# Supplementary figures and images for: Silencing of XB130 Is Associated with Both the Prognosis and Chemosensitivity of Gastric Cancer
Source: PLoS One. 2012 Aug 23;7(8):e41660. doi: 10.1371/journal.pone.0041660 (PMC3426513; doi:10.1371/journal.pone.0041660)

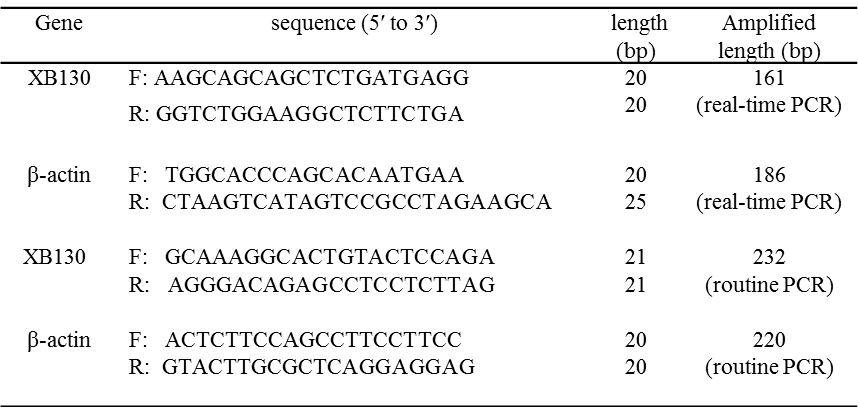


**Table S1.** Primer sequences for Real-time or routine PCR

Supplement: Table S1 — Primer sequences for Real-time or routine PCR. (DOC) [file pone.0041660.s002.doc]

**Table S2.** XB130 Sh-RNA sequences


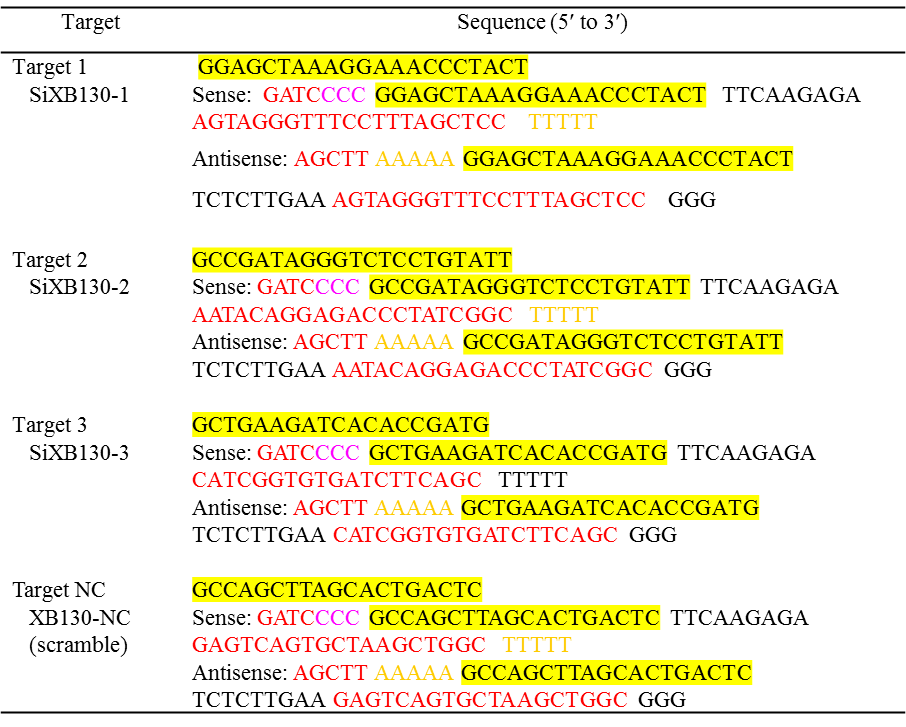

Supplement: Table S2 — XB130 Sh-RNA sequences. (DOC) [file pone.0041660.s003.doc]
